# Bench-to-Bedside and Bedside Back to the Bench; Seeking a Better Understanding of the Acute Pathophysiological Process in Severe Traumatic Brain Injury

**DOI:** 10.3389/fneur.2015.00047

**Published:** 2015-03-17

**Authors:** Denes V. Agoston

**Affiliations:** ^1^Department of Anatomy, Physiology and Genetics, Uniformed Services University, Bethesda, MD, USA; ^2^Department of Neuroscience, Experimental Neurotrauma, Karolinska Institutet, Stockholm, Sweden

**Keywords:** traumatic, brain, injury, acute, severe

## Abstract

Despite substantial investments, traumatic brain injury (TBI) remains one of the major disorders that lack specific pharmacotherapy. To a substantial degree, this situation is due to lack of understanding of the pathophysiological process of the disease. Experimental TBI research offers controlled, rapid, and cost-effective means to identify the pathophysiology but translating experimental findings into clinical practice can be further improved by using the same or similar outcome measures and clinically relevant time points. The pathophysiology during the acute phase of severe TBI is especially poorly understood. In this Mini review, I discuss some of the incongruences between current clinical practices and needs versus information provided by experimental TBI research as well as the benefits of designing animal experiments with translation into clinical practice in mind.

Traumatic brain injury (TBI) annually affects more than 10 million people worldwide (1–3). Changes in life styles and in demographics, such as increased motorization and an aging population, will likely render TBI the number one cause of disability and death by 2020 (4). The direct and indirect costs of TBI are enormous. In the year 2000, the cumulative cost of medical treatment, rehabilitation, lost productivity, and so forth was approximately 60 billion dollars in the United States alone (4–6). Individuals who have suffered a TBI are far more susceptible to developing chronic neurodegenerative conditions, such as Alzheimer’s Disease (AD) which further increases the medical, financial, societal, and emotional burdens of TBI (7–9). TBI is not a single disease but a spectrum of disorders currently classified as severe, moderate, or mild and TBI is the only major disease that has no specific pharmacotherapy (10, 11).

Epidemiology data show that early intervention is a key determinant of favorable outcome in severe TBI (12–16). Neuromonitoring, structural imaging, and other diagnostic techniques employed in a modern neurointensive care unit (NICU) provide real-time or near real-time information about changes in cerebral metabolism, cerebral blood flow (CBF), and intracranial pressure (ICP) among other parameters (10, 17, 18). The collected data guide available interventions that are aimed at normalizing the intracerebral milieu in support of recovery. Functional outcome can be further improved, if we know the identity, onset, and extent of the specific pathologies associated with the secondary injury process, by means of targeted, evidence-based pharmacotherapy. Experimental TBI can provide this much needed information; however, most experiments are not designed to deliver clinically relevant data. In clinical settings, the early, acute post-injury phase is marked by rich data collection using various modalities/outcome measures. Importantly, the measurements are frequently repeated in NICUs resulting in a high temporal resolution. Experimental TBI studies have not focused on this acute post-injury period and typically use clinically irrelevant outcome measures such as histology.

In this Mini review, I focus on severe TBI that requires neurointensive care, list the incongruences between experimental and clinical TBI research, discuss some of the issues that underlie the current gap, and outline potential solutions that can narrow the gap with benefits for both clinical and experimental TBI fields.

## Neurological Evaluation

The Glasgow Coma Scale (GCS), length of loss of consciousness (LOC), alteration in mental/conscious sate (AOC), and post-traumatic amnesia (PTA) are the “gold standards” of determining injury severity in clinical care (10). These tests are used worldwide due to their low-tech requirements and ease of administration. Practically all clinical TBI reports contain GCS (as well as LOC, AOC, and PTA) data, thus enabling comparisons between studies. These assessments are used for triaging and selecting patients for clinical trials, and in epidemiology studies. Their modified versions, such as the Glasgow Outcome Scale (GOS), are used to assess the outcome and the efficacy of drug treatments in clinical trials. The tests are typically repeated over a period of time, thereby providing critical information about the temporal profile of functional changes. While the subjective nature of these tests and the impact of pre-hospital care are important confounding factors, they will not be discussed in this paper.

The lack of comparable data in experimental TBI represents one of the major gaps between the two fields. Despite the availability of inexpensive neurobehavioral and injury severity tests for rodents (19), a PubMed search shows that only a fraction of experimental TBI studies utilize such tests. This phenomenon creates the following paradox: in clinical TBI, we know the extent of the functional deficits but not the parameters of the causative physical forces, and in experimental TBI, we know the parameters of the physical forces (we can calibrate them) but (typically) not the type and extent of the functional deficits caused by these forces. The temporal aspect of injury severity assessment is also overlooked in experimental vs. clinical TBI. Even though the similarities and/or dissimilarities between the timelines of human and rodent pathologies (in severe TBI or in any other conditions) are not known, assessing injury severity at several early time points in experimental TBI could provide important, albeit currently missing information that mimics clinical practice. Additionally, serial assessment of neurological deficits would greatly benefit the experimental TBI field by validating existing models and helping to determine the correlation between physical forces and the biological/functional responses to them.

## Structural Imaging

Structural imaging by computed tomography (CT) and magnetic resonance imaging (MRI) or diffusion tensor imaging (DTI) is routinely performed upon admission to a NICU, and the pathoanatomic information is quantified by scores, such as Marshall and Rotterdam (20–22). Imaging can identify a range of macroscopic changes that include skull fracture, contusion, laceration, hemorrhage of different types, swelling/edema, as well as axonal injury (10). Furthermore, serial imaging can provide information about disease progression and the efficacy of interventions in an objective manner. Contrary to its routine use in clinical settings, there are no experimental TBI studies using CT imaging. A PubMed search of existing literature indicates that imaging, typically MRI or positron emission tomography (PET) studies, is rarely performed in experimental TBI. Importantly, very few if any of these studies focused on structural changes during the clinically relevant acute post-injury period.

*In vivo* imaging of small animals presents a number of technical challenges, including low spatial resolution and dealing with motion artifacts (23). Obtaining sufficient spatial resolution of the rat brain requires several hours of scanning time, which can only be performed under anesthesia. This may not be a major caveat given that most TBI patients in NICUs are sedated; however, the limited availability of imaging modalities optimized for small animal research makes scanning live animals very difficult. The alternative is to perform *ex vivo* imaging. In addition to flexible scheduling, *ex vivo* imaging allows for extended scanning times. Nonetheless, the fixation process can produce artifacts that may affect imaging quality (e.g., reduced DTI sensitivity) (24). A major unresolved issue in animal imaging studies is the lack of standard analytical protocols, including standardized brain atlases for rodents (25–28). In the absence of such standards, imaging studies performed at different laboratories are rendered incomparable.

The benefits of performing imaging studies in experimental TBI include the validation of TBI models and the elucidation of injury heterogeneity, which is inherent in human TBI. Analyzing and interpreting imaging data, a routine in clinical setting, in the context of histological changes, a major experimental outcome, would allow us to relate structural level changes to cellular level changes. For example, we currently have a limited understanding of how altered fractional anisotropy (FA) values relate to axonal function. Experimental TBI studies that integrate MRI and histopathology can address this important issue. Combined with yet another clinically relevant non-invasive technique that measures axonal functionality, magnetoencephalography (MEG), we can gain an even deeper understanding of the connection between structural changes and altered functionality (29, 30).

## Physiological/Systemic Monitoring

Physiological parameters such as blood pressure, breathing, heart and pulse rates, and blood oxygen saturation are monitored in the NICU. Injury to the head alone can adversely affect vital physiological functions, e.g., by causing apnea. Apnea contributes to decreased blood oxygen saturation which in turn triggers complex downstream molecular events, including the activation of hypoxia-induced molecular compensatory mechanisms (31). However, severe head injury without additional trauma is very rare. Polytrauma, a frequent occurrence in severe human TBI cases, can substantially complicate the course and outcome of the brain injury but rarely modeled in experimental TBI.

The monitoring of vitals, such as heart rate, breathing rate, and oxygen saturation, is seldom performed in experimental TBI despite the availability of devices (and analysis software) specifically adapted for use in rodents (32, 33). Monitoring such parameters in experimental TBI and analyzing the data in the context of histological and molecular (and functional) outcomes would help clarify the role the secondary injury process plays in affecting systemic pathological changes. This knowledge would provide the basis for improving current NICU practices and making TBI models more clinically relevant.

## Cerebral Monitoring

In the absence of specific, evidence-based therapeutic interventions in severe TBI, the current strategy is to normalize the intracerebral environment, thereby facilitating recovery (“*medicus curat, natura sanat*” meaning the physician treats, nature heals). The anatomical and biochemical uniqueness of the central nervous system (CNS) requires close monitoring of ICP, cerebral perfusion pressure (CPP), CBF, and cerebral metabolic rate of oxygen (CMRO_2_), and glucose consumption (CMRGlc) (34, 35). Early experimental studies have determined the effects of cerebral insult(s) in various animal models of TBI; however, these studies are far from being clinically relevant (36, 37). Epidemiology studies have shown that monitoring and maintaining these parameters within their normal physiological ranges is a key determinant of functional outcome (10, 18). Transcranial Doppler (TCD) (38, 39) and quantitative electroencephalography (qEEG) (40) have been used in clinical settings to provide information about blood flow, seizure activity, ischemia, and vasospasm. All of these monitoring techniques currently have animal versions available (41). Combining the readouts of TCD and qEEG measurements with biochemical, molecular, and histological analyses in experimental TBI studies can help interpret clinical TCD and qEEG data in the context of molecular and cellular pathologies that are easily measurable under controlled experimental conditions. Experimental TBI would also benefit from such analyses, particularly when the validation of experimental models is concerned. A limited number of TBI studies using large animal models have provided information about changes in the intracranial cellular environment over time in a setting closely mimicking NICU (42). Using the controlled cortical impact (CCI) model of swine, this pioneering study has established the correlation between injury severity (the depth of depression), ICP (as well CPP) and neuronal degeneration, axonal damage, and cell death. The obtained data provided the basis to test an evidence-based therapeutic measure, infusing hemoglobin-based oxygen-carrying (HBOC) solution after severe TBI with hemorrhage (43). HBOC treatment improved CPP and MAP and significantly improved histological outcome. These studies are great examples of how experiments designed to mimic clinical scenarios can result in developing evidence-based treatments in TBI and can also provide information about the correlation between physical forces and tissue damage thereby helping to calibrate and validate experimental TBI models.

## Biochemical Monitoring of Biosamples

In a typical NICU, systemic blood is routinely collected (as frequently as necessary) for monitoring changes in electrolytes, metabolites, and inflammatory markers (15, 44–47). Serum levels of various protein biomarkers indicative of neuron and/or glia damage and/or loss –neuron-specific enolase (NSE), neurofilament (NF), tau protein and its phosphorylated forms, ubiquitin carboxyl-terminal hydrolase-L1 (UCH-L1) and αII-spectrin breakdown product (SBDP145), glial fibrillary acidic protein (GFAP), and S100β – are also getting measured (48–52). It has been shown that the temporal profile (or trend) of changes in serum biomarker levels may be an important indicator of outcome (53, 54). However, the reported associations between serum levels of these protein biomarkers and injury severity and/or outcome are rather study specific. Despite the clinical practice and importance of such monitoring, obtaining and analyzing blood/serum samples at multiple post-injury time points (monitoring), are almost completely absent in experimental TBI work. While obtaining serial blood samples from small rodents under anesthesia can be challenging, determining time-dependent changes in serum biomarker levels has a very high clinical relevance. Serum biomarker data obtained in an experimental setting, when analyzed and interpreted in the context of other outcome measures (e.g., histology), greatly facilitate the understanding of serum biomarker changes as they relate to specific pathologies, injury severity, and prognosis.

Analyzing biomarkers in the cerebrospinal fluid (CSF) significantly improves the CNS specificity of detected changes (10, 55–59). CSF is also routinely collected in NICUs and is analyzed for changes in metabolism, inflammation, and the presence of infectious agents. Similar to blood/serum samples, CSF has been increasingly tested in clinical TBI for changes in protein biomarkers (56, 60–64). There are several promising candidates including ubiquitin C-terminal hydrolase-L1 (UCH-L1) that show correlation with injury outcomes. In contrast to clinical practice, very few experimental TBI studies have analyzed injury-induced changes of biomarkers in the CSF (65–67). Obtaining CSF from small rodents is not without its technical challenges, but volumes (~50 μl) sufficient for several biochemical and immunological assays can be obtained from rats using cistern puncture. Even though obtaining CSF from large animals (e.g., pigs) is relatively easy, it also rarely performed (65). Experimental data obtained by analyzing CSF samples at multiple time points, mimicking clinical practice, would be especially valuable in validating clinically used biomarkers and linking their changes to injury severity and outcome. Concurrent sampling and subsequent analysis of serum and CSF samples would help validate injury-induced changes in blood-based biomarkers in the context of injury severity as well as CNS specificity.

The CNS specificity of biomarkers can be even further improved by analyzing injury-induced changes in the brain interstitial fluid (ISF) collected by cerebral microdialysis (CMD) (68–73). The biochemical analysis of ISF has been used to monitor cerebral metabolism in numerous NICUs, because it provides important spatial information about ongoing pathological changes (10). Novel types of microdialysis catheters enable the collection of molecules up to 100 kDa molecular weight. Studies have shown that ISF samples can be analyzed by high-resolution mass spectrometry-based proteomics (74–76). Performing similar analyses in experimental TBI would require the use of large animal models with gyrencephalic brain and physiology close to humans.

## Caveats

Important, albeit potentially confounding factors such as pre-existing and/or predisposing conditions (medications, the role of age and gender), comorbidities (polytrauma), and the effect of anesthesia are beyond the scope of this paper. While we are seeking to match clinical and experimental practices and outcomes, it is essential to remember that humans and rodents (most frequently used in TBI modeling), live on very different timescales (77). Therefore, scalability – due to innate species differences in anatomy and physiology – is of the utmost importance when it comes to animal modeling of a complex human disease. For example, protein turnover and metabolic rate, respectively, are ~10× and ~6× higher in rats compared to humans (78, 79). Heart and respiration rates are ~4–6× higher in the rat while gestation and sexual maturity, respectively, are ~12× and ~100×, faster. Of the pathological processes after various insults including TBI, inflammation (various components) is 4–30× faster in the rat (80). There is no algorithm or “conversion formula” available, even for normal biological processes. The lack of such information represents an important gap in our understanding of how the temporal profiles of clinical and experimental TBI data compare. Furthermore, complex pathological processes such as inflammatory response can be vastly different in rodents than in humans (80).

Finally, the rapid advancement of non- and/or minimally invasive technologies, various imaging modalities combined with increasingly powerful analytical tools, finite element, and other computational modeling will eventually make experimental TBI obsolete (81). In the meantime, TBI experiments should be designed with real-life clinical TBI in mind. Doing so would not only provide clinicians with much needed data to refine current clinical practices, but also help research scientists validate and refine their experimental models. An additional benefit for the clinical TBI field would be the ability to analyze clinical data in the context of molecular pathologies studied (and validated) under controlled conditions – a fundamental step toward targeted, evidence-based pharmacotherapies for TBI.

## Conflict of Interest Statement

The author declares that the research was conducted in the absence of any commercial or financial relationships that could be construed as a potential conflict of interest.
